# How frequently are insects wounded in the wild? A case study using *Drosophila melanogaster*

**DOI:** 10.1098/rsos.240256

**Published:** 2024-06-26

**Authors:** Bengisu S. Subasi, Veit Grabe, Martin Kaltenpoth, Jens Rolff, Sophie A. O. Armitage

**Affiliations:** ^1^ Institute of Biology, Freie Universität Berlin, Berlin, Germany; ^2^ Microscopic Imaging Service Group, Max Planck Institute for Chemical Ecology, Jena, Germany; ^3^ Department of Insect Symbiosis, Max Planck Institute for Chemical Ecology, Jena, Germany

**Keywords:** cuticle damage, cuticle injury, cuticle wound, melanin, mite, wild-collected organism

## Abstract

Wounding occurs across multicellular organisms. Wounds can affect host mobility and reproduction, with ecological consequences for competitive interactions and predator–prey dynamics. Wounds are also entry points for pathogens. An immune response is activated upon injury, resulting in the deposition of the brown-black pigment melanin in insects. Despite the abundance of immunity studies in the laboratory and the potential ecological and evolutionary implications of wounding, the prevalence of wounding in wild-collected insects is rarely systematically explored. We investigated the prevalence and potential causes of wounds in wild-collected Drosophilidae flies. We found that 31% of *Drosophila melanogaster* were wounded or damaged. The abdomen was the most frequently wounded body part, and females were more likely to have melanized patches on the ventral abdomen, compared with males. Encapsulated parasitoid egg frequency was approximately 10%, and just under 1% of Drosophilidae species had attached mites, which also caused wounds. Wounding is prevalent in *D. melanogaster*, likely exerting selection pressure on host immunity for two reasons: on a rapid and efficient wound repair and on responding efficiently to opportunistic infections. Wounding is thus expected to be an important driver of immune system evolution and to affect individual fitness and population dynamics.

## 1. Introduction

Wounding is widespread among wild organisms [1]. Wounds have been documented in wild vertebrates such as lizards [2] and snakes [3], invertebrates such as crustaceans [4], a variety of benthic invertebrates [5] and insects [6–8]. Wounds can potentially have ecological consequences for predator–prey dynamics, population dynamics and competitive interactions [1], and they can have evolutionary consequences through their effect on fitness and the selection pressure they impose on immune defences [4]. Field studies provide vital insight into the importance of selective pressures under natural conditions (e.g. [9]); therefore, to estimate the potential impact of wounding, systematic wild-collected quantitative data are required on their occurrence. Even though there are a number of reports of wounds in wild insects, there is a paucity of studies that have systematically surveyed the extent of wounding in the wild (but see e.g. [8]). We, therefore, systematically investigate the prevalence and potential causes of wounding in *Drosophila melanogaster*, an insect that is abundantly found in the field, and commonly used as a laboratory model.

A variety of body parts have been found to be wounded in wild-collected insects, including wing damage [10,11], loss of antennae or legs [8,12] and abdominal wounds [7]. For example, around 30% of adult bush crickets, *Decticus verrucivorus*, had antennal or leg damage [7]. Furthermore, laboratory studies show that intra- and extragenital wounds are found across species [13,14]. Studies on marine benthic invertebrates [5] and the crustacean *Gammarus pulex* [4] have shown that wounding rates can differ temporally, geographically and according to sex, but these factors are relatively unexplored in insects. Damage can occur for a variety of reasons, including predator–prey interactions [12], intra- and inter-specific competition over food, territory or mating [15] or wear and tear caused by environmental factors [16]. In some species, intersexual conflict results in copulatory wounds, particularly to the females [14,17]. Furthermore, parasites such as mites can wound the host cuticle with their mouth parts (chelicerae) when feeding on haemolymph [18]. Wounds can be costly, for example by reducing survival [8,19] including for *D. melanogaster* [20], enhancing susceptibility to attack by predators [21] or pathogens [12], or by negatively affecting reproduction [22,23].

Importantly, wounds can be entry points for infections. Organisms are constantly exposed to microbes in their environment, and the insect cuticle, including in *D. melanogaster*, can harbour microbial communities [24,25]. Once the cuticle is breached, an opportunistic pathogen could potentially enter the body. For example, in a non-sterile environment, the mortality of wounded ants was 80% in 24 h while it was only 10% in a sterile environment [12]. Similarly, after a leg wound in *D. melanogaster*, the spread of bacteria into the fly’s body and the pathogenicity of the bacteria affected fly survival [26]. Furthermore, parasitic mites puncture the host cuticle and can act as disease vectors in honeybees and increase the hosts’ susceptibility to viral [27] and bacterial infections [18]. Mites have also been experimentally shown to transmit *Spiroplasma poulsonii*, a male-killing endosymbiont of *Drosophila nebulosa* and *Drosophila willistoni*, from infected to uninfected flies, both within and between different species of *Drosophila* [28].

In insects, wounding induces an immune response similar to that used to encapsulate parasites and pathogens. It has long been recognized that wounding in insects can result in brown to black melanized marks on the cuticle, for example topical scratching and abrasion induced by conspecifics in the silkworm *Bombyx mori* (Pasteur 1870, referenced in [29]) and experimentally scratched *D. melanogaster* larval cuticle [30]. Melanin is a pigment that plays a central role in insect immunity and cuticular darkening [31]. If the wound is more severe than a scratch, and the epidermis and basement membrane underlying soft cuticle are breached, haemolymph coagulation and clotting rapidly occur, thus preventing both haemolymph loss and microorganisms from passing into the haemocoel [32–34]. The prophenoloxidase cascade is also immediately activated leading to melanization and a hard clot [33], which is observable as brown to black pigmentation. It is thought that coagulation and clotting do not occur in insects with a hard cuticle, because their haemolymph is under less than atmospheric pressure [33]; nonetheless, wounds in individuals with hard cuticles are still melanized, including adult *D. melanogaster* (e.g. [35]). The melanized wounds within a larval instar or within the pupal or adult phase remain visible on the cuticle for that life stage, and as such, they are a signature of wounding during that phase. Wounding, therefore, exerts two concurrent selection pressures on the immune system: first on a rapid and efficient wound repair, and second on responding to microbial invaders. Furthermore, endoparasitoids lay their eggs on or in the *Drosophila* body during the larval or pupal life history stages [36]. The presence of wasp eggs triggers a haemocyte-mediated encapsulation reaction and if the immune response is successful the parasitoid egg is encapsulated and can be seen as a melanized black area under the cuticle of all subsequent life stages [37].

In the wild, *D. melanogaster* is found on fermenting fruits and other plant material: these resources also attract other drosophilids as well as other arthropods and microbes [38]. *D. melanogaster* is associated with many bacteria [39], and yeast species [40], which are nutritionally important. Furthermore, several naturally occurring parasites and pathogens have been documented (reviewed in [41]). *D. melanogaster* has been widely used in studies of insect immunity [42] and as an invertebrate model for wound healing in the labratory [34,43]. Experimental evidence from laboratory studies shows that copulatory wounds are found across many female *Drosophila* species including the melanogaster sub-group [13,44], and wing damage in *D. melanogaster* has also been noted in the laboratory [45]. There is anecdotal evidence of darkened melanized spots on the cuticle of wild-collected *D. melanogaster* [46]. However, there is no systematic study exploring the prevalence of wounding in wild *D. melanogaster*, and thus its potential ecological and evolutionary significance is unknown.

In this study, we determined the frequency and type of wounds that female and male *D. melanogaster* sustain in the wild. We systematically collected adult flies across three seasons and three locations and examined them for melanized areas on the cuticle which we use as a proxy for wounding, and the presence of melanized patches under the abdomen as support for parasitoid egg encapsulation. Lastly, as mite mouthparts can potentially penetrate and wound the host cuticle, we collected more than 8000 flies, including other Drosophilidae species, to assess the frequency with which mites are found.

## 2. Material and methods

### 2.1. Sampling sites and collection methods

Adult flies were collected from three farms in and around Berlin, Germany: Domaene Dahlem (D), Obsthof Lindicke (L) and SL Gartenbau (G), located approximately 30–40 km apart. Permission was obtained from landowners to conduct the fieldwork detailed in this study, and no other fieldwork permissions were required. Collections were carried out between June and October 2021 in early summer (ES, 24 June–21 July), late summer (LS, 24 August–6 September) and autumn (A, 4–8 October). The flies were collected using traps; details are in electronic supplementary material, S1. We captured several Drosophilidae species, but we aimed to examine only *D. melanogaster* for wounds. Except for *D. simulans* females, both sexes from all other species could be distinguished from *D. melanogaster* based on their morphology. To distinguish between *D. melanogaster* and *D. simulans* females after examining them for wounding, we carried out a diagnostic PCR (see electronic supplementary material, S1). All captured individuals and species were examined for the presence of mites.

### 2.2. Examination methods for cuticular damage

**Figure 1.**
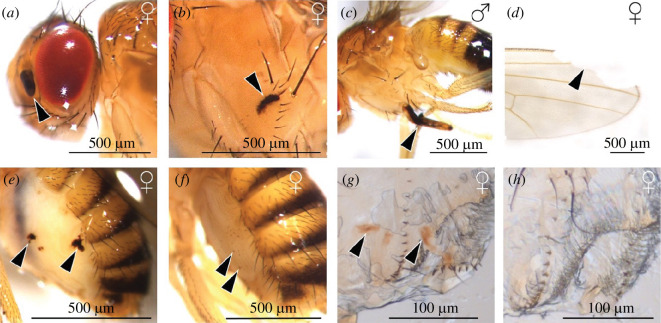
Examples of wounding and damage in *D. melanogaster*. Arrows indicate melanized areas likely resulting from an immune response or missing parts of the wings. Wild-collected flies with damage to the (*a*) head, (*b*) thorax, (*c*) leg, (*d*) wing, (*e*,*f*) ventral abdomen. Images from labratory-reared flies under controlled mating conditions to illustrate (*g*) wounds on the female vaginal furcal dorsolateral fold (formerly termed lateral folds in [44], more recent terminology from [47]) resulting from mating and (*h*) a virgin female without copulatory wounds. The sex of each fly is indicated at the top right of each image. Images (*g*,*h*) taken by A. Finsterbusch.

We examined a total of 1246 flies for damage: 638 *D. melanogaster* males and 608 females (the latter number contains *D. melanogaster* and *D. simulans*). Flies were examined blind and randomly with respect to season and site; for sample sizes, see electronic supplementary material, table S1. The whole body was carefully examined for melanized spots (figure 1; electronic supplementary material, S1 and S2), which are an indicator of past damage or to check for missing body parts (e.g. legs or parts of wings; figure 1*c*,*d*, respectively). It is important to note that it was not possible to discriminate between wounds that would probably have penetrated the cuticle and wounds that led to melanization owing to abrasion or scratches on the exterior surface of the cuticle, or melanized spots that may appear on the cuticle owing to internal physiological responses. We also note that we mostly use the term ‘wound’ but that some literature uses the term ‘injury’ and others ‘damage’ to refer to similar phenomena. We also recorded large, melanized areas under the cuticle, which are indicative of encapsulated parasitoid eggs (electronic supplementary material, S1). The flies were examined under a Leica M205C stereomicroscope at up to 160× magnification. Images were captured with a Leica FLEXACAM C1 and Leica Application Suite.

### 2.3. Mites

In total, 8019 flies (7612 *D. melanogaster*/*D. simulans*, 407 other Drosophilidae) were examined for the presence of mites. We recorded the number of attached mites, which part of the fly body they were attached to, as well as the season and collection site. The species of flies and the mites were identified using molecular methods (electronic supplementary material, S1), or they were identified morphologically by Darren Obbard by using photographs. Where possible, we determined the sex of the flies. We also removed the mites to see whether melanized spots were present where the mite mouthparts had been. To examine the attachment of mites to the fly we used scanning electron microscopy (SEM) and X-ray microtomography (µCT), and the latter was also used to examine encapsulated parasitoid eggs (electronic supplementary material, S1).

### 2.4. Statistical analyses

Analyses were performed using R v. 4.2.2 [48] in RStudio v. 2022.07.2. Details of all models are in electronic supplementary material, S1.

## 3. Results

In total, 1246 flies were examined for wounding or damage, of which 638 were morphologically identified as male *D. melanogaster*. Diagnostic PCRs allowed us to identify 536 female *D. melanogaster* and the remaining 71 females were *D. simulans*. The latter were analysed separately from *D. melanogaster* (for results, see electronic supplementary material, S3).

### 3.1. Comparisons between head, leg, thorax and abdomen damage

**Figure 2.**
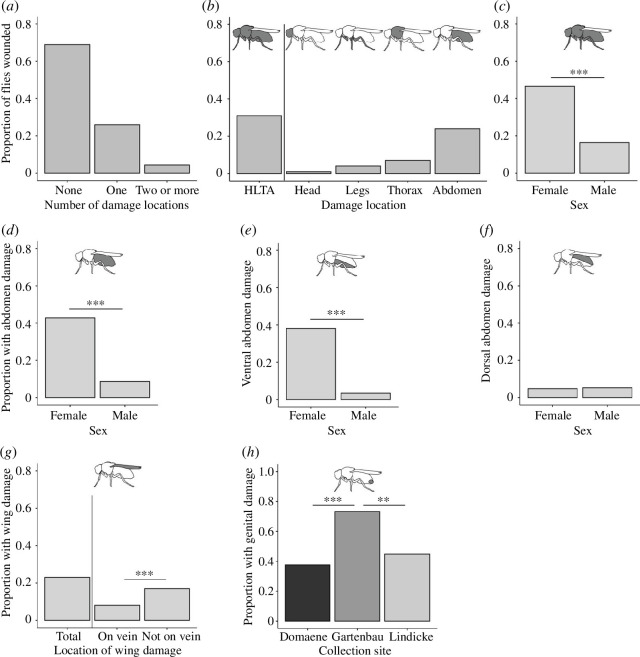
Frequency of wounding in wild-collected *D. melanogaster*. (*a*) The proportion of flies with none, one or two or more damage locations, (*b*) the proportion of flies with wounds to the external cuticle (HLTA: head, legs, thorax and abdomen), (*c*) the proportion of females and males showing HLTA wounding, (*d–f*) the proportion of the flies with total, ventral and dorsal abdominal melanized spots by sex (for *a–f*, *n* = 1174), (*g*) the proportion of flies with wing damage (total) and damage that is either to a vein or not to a vein (*n* = 338), and (*h*) the proportion of females showing copulatory wounding according to collection site (*n* = 163). Stars indicate statistically significant differences where: **p <0.001 and ***p <0.0001. The grey-shaded areas of the cartoon flies show the area of damage in each panel.

Flies differed significantly in the total number of body parts that were wounded (*χ* ^2^ = 769.81, d.f. = 2, *p* < 0.0001, *n* = 1174); they most frequently showed wounds on only one body part, with a small percentage of the flies having two or more wounded body parts (figure 2*a*). In total, 31% of individuals showed at least one type of wound on the external cuticle (figure 2*b*). The head, legs, thorax and abdomen differed significantly in the frequency with which they were wounded (*χ* ^2^ = 437.09, d.f. = 3, *p* < 0.0001, *n* = 1174), with the abdomen most frequently showing damage (figure 2*b*).

Next, we analysed the frequency of wounding while controlling for the relative area of the head, thorax and abdomen using a proxy for their relative sizes. This analysis was carried out separately for females and males as they differed slightly in the relative sizes of their body parts (electronic supplementary material, S1). For females, the abdomen was still more frequently damaged compared with the head and the thorax, although the head and thorax no longer differed significantly after controlling for body part area (electronic supplementary material, table S2). However, for males, the abdomen showed a similar frequency of wounding compared to the thorax, both with and without the correction for relative body part area (electronic supplementary material, table S2).

### 3.2. Combined damage across head, leg, thorax and abdomen

When we combined damage across the head, thorax, legs and abdomen (not including copulatory wounds), we found that females were more frequently injured than males (electronic supplementary material, table S3; figure 2*c*). There was also a significant interaction between site and season (electronic supplementary material, table S3), which was owing to late summer differences, where flies from the D site were less frequently damaged compared with those from the G site (Tukey *post hoc* test, *z* = −4.260, *p* = 0.0007) and the L site (*z* = −4.356, *p* = 0.0005); furthermore flies from the G site in late summer were more frequently damaged compared with flies from the L site in the early summer (Tukey *post hoc* test, *z* = −3.266, *p* = 0.030).

### 3.3. Thorax and abdomen damage

Season and collection site significantly affected the proportion of flies with thorax damage (electronic supplementary material, table S4). *Post hoc* multiple comparisons showed that the early summer flies less frequently had damage to their thoraces compared with late summer flies (Tukey *post hoc* test, *z* = −2.82, *p* = 0.0134).

Females were significantly more likely to have damage on their abdomens compared with males (electronic supplementary material, table S5; figure 2*d*). There was also a significant interaction between season and site; after multiple comparisons, late summer flies from site D had significantly more damage on their abdomen compared with those from sites G (Tukey *post hoc* test, *z* = −3.154, *p* = 0.0131) and L (Tukey *post hoc* test, *z* = −3.690, *p* = 0.0069). When we considered the ventral and dorsal abdomen separately, we found that the ventral abdomen was more frequently wounded compared with the dorsal abdomen (*χ* ^2^ = 96.50, d.f. = 1, *p* < 0.0001, *n* = 1174; figure 2*e,f*). Furthermore, females were more likely to have damage to their ventral abdomen compared with males (electronic supplementary material, table S5) and there was a significant interaction between season and site. After multiple comparisons, it was revealed that early summer flies from site D had significantly more damage than late summer flies from site D (Tukey *post hoc* test, *z* = 3.116, *p* = 0.0480), and late summer flies from site D had more damage than late summer flies from sites G (Tukey *post hoc* test, *z* = −3.321, *p* = 0.0252) and L (Tukey *post hoc* test, *z* = −4.004, *p* = 0.0020). However, there was no significant effect of any factors on the damage to the dorsal abdomen (electronic supplementary material, table S5).

### 3.4. Wing damage

In total, 338 flies (165 females and 173 males) were examined for wing damage. Damage was more frequently found to affect an area of the wing without veins than an area with veins (*χ* ^2^ = 12.81, d.f. = 1, *p* = 0.0003; figure 2*g*). When we only considered wing damage that affected veins, we found a significant difference between sites (electronic supplementary material, table S6) but after multiple comparisons, these differences no longer remained significant. Season was the only factor to affect the wing damage not on the veins (electronic supplementary material, table S6) and multiple comparisons showed that the proportion with wing damage was significantly higher in autumn compared with late summer (Tukey *post hoc* test, *z* = −2.860, *p* = 0.0118).

### 3.5. Copulatory wounds

In total, 178 females were examined for copulatory wounds, and of those 163 were molecularly identified as *D. melanogaster* and 15 as *D. simulans*. Fifty-two per cent of *D. melanogaster* showed copulatory wounds, and the proportion varied significantly with the collection site (electronic supplementary material, table S7; figure 2*h*). We hypothesized that abdominal damage found on females might be related to mating, and there was a marginally non-significant positive relationship (LR Chisq = 3.74, *p* = 0.0527) between the presence of both copulatory and abdominal wounds. When focusing only on damage to the ventral abdomen, there was also no significant correlation.

### 3.6. Encapsulated melanized parasitoid eggs

**Figure 3.**
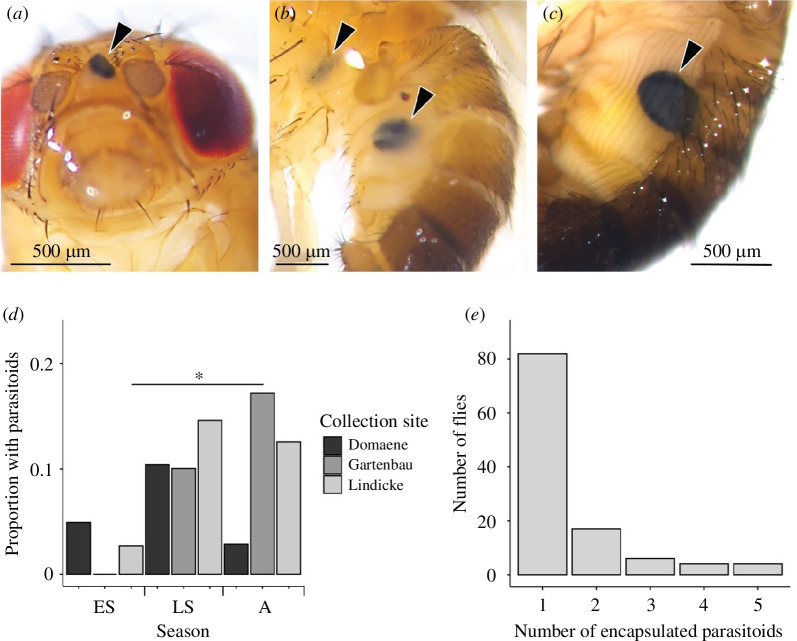
Melanized encapsulated parasitoid eggs in wild-collected *D. melanogaster*. Images of melanized encapsulated parasitoid eggs in (*a*) the head, (*b*) the thorax and abdomen, and (*c*) the abdomen, (*d*) the proportion of flies with parasitoids by season and site and (*e*) the proportion of flies with one or more encapsulated parasitoids (for *d*, *n* = 1174 flies were examined). ES: early summer, LS: late summer and A: autumn. The asterisk indicates *p* < 0.05 and the line indicates the significant *post hoc* difference between the early summer Lindicke site and the late summer Gartenbau site.

Melanized parasitoids were predominantly observed in the abdomen, with one found in the head and another in the thorax (figure 3*a–c*). A melanized parasitoid egg was visible through the cuticle of 9.7% (114 out of 1174) flies. Season and site together affected the proportion of flies with parasitoids (electronic supplementary material, table S8; figure 3*d*), which was driven by differences between the early summer L site and late summer G site (Tukey *post hoc* test, *z* = −3.22, *p* = 0.0346). There was also a significant interaction between sex and site (electronic supplementary material, table S8) although after multiple comparisons none of the groupings differed from each other. Flies differed significantly in the number of encapsulated parasitoid eggs that they exhibited (*χ* ^2^ = 200.32, d.f. = 4, *p* < 0.0001, *n* = 1174, figure 3*e*). The interaction between season and site significantly affected the number of encapsulated parasitoid eggs (electronic supplementary material, table S8).

### 3.7. Mites

**Figure 4.**
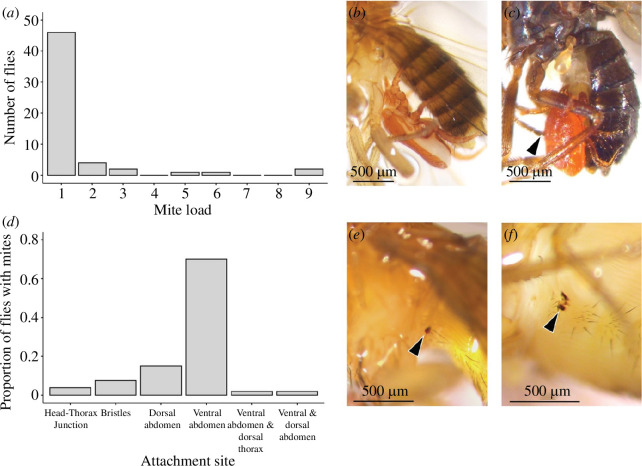
Mites found on wild-collected Drosophilidae. (*a*) Mite load across all flies, (*b*) *Macrocheles* sp. attached to a female *D. melanogaster*, (*c*) a *Pergamasus* sp. attached to a *D. subobscura*, (*d*) the numbers of mites attached to different body parts. The bristles were on the head and the thorax. One fly had morphologically similar mites attached to the ventral abdomen and to the dorsal thorax, and another fly was found with mites attached to the ventral and the dorsal abdomen. The melanized areas visible on the abdomen of (*e*) a male *D. melanogaster* and (*f*) a female *D. melanogaster,* after removing the mites. For (*a*,*d*), *n* = 56.

In 0.7% (56 of 8019) of the collected flies, one or more mites were attached to the fly body (figure 4*a*). Mites were found on seven Drosophilidae species: *Drosophila busckii*, *Drosophila hydei*, *D. melanogaster, D. simulans* and *Drosophila subobscura* which were identified via sequencing (per cent matches for all sequencing results can be found in electronic supplementary material, table S9), and *Drosophila funebris* and *Scaptomyza pallida*, which were identified morphologically. We identified 18 out of 56 mites via sequencing and the rest remained unidentified; two mites were identified to the genus level: *Macrocheles* sp. (figure 4*b*) and *Pergamasus* sp. (figure 4*c*), and one mite had a closest match to *Archidispus insolitus*. Among the identified mites, 14 of the flies were found to be parasitized with *Macrocheles* sp. while *Pergamasus* sp. was found on two *D. melanogaster* and one *D. subobscura*; *A. insolitus* was found on one *D. subobscura* (electronic supplementary material, table S9).

There was significant variation in the number of mites that were attached to a fly (*χ* ^2^ = 173.5, d.f. = 5, *p* < 0.0001, *n* = 56; figure 4*a*), and their attachment sites (*χ* ^2^ = 111.72, d.f. =5, *p* < 0.0001, *n* = 53; figure 4*d*), together indicating that flies that were carrying mites tended to only have a single attached mite and that the ventral abdomen was the dominant attachment site. After removing the attached mites, 74.4% (32 of 43 flies) had melanized patches: significantly more flies had melanized patches compared with no melanized patches (*χ* ^2^ = 9.30, d.f. = 1, *p* < 0.0023, *n* = 43; figure 4*e,f*). We observed melanized patches on all flies infested with *Macrocheles* sp. and two out of three flies infested with *Pergamasus* sp. The *A. insolitus* was found detached from the fly, and no melanized patches were observed on the fly. Sex, season and site had no significant effect on the probability of having an attached mite (electronic supplementary material, table S10). For µCT images of the parasitoids eggs, see electronic supplementary material, S4.

**Figure 5.**
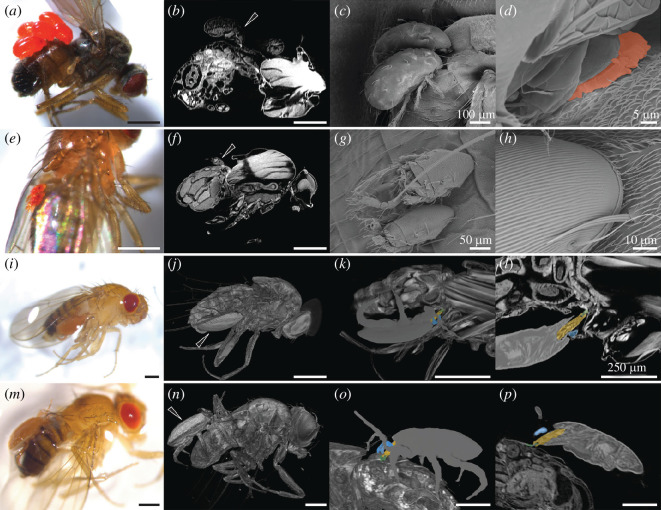
µCT and SEM of mites attached to Drosophilidae. (*a–c*) *Scaptomyza pallida* with attached mites whose chelicerae appear to slightly penetrate the dorsal abdominal cuticle and (*d*) where a mite- or fly-derived substance (orange) is visible at the contact point. (*e–h*) Two smaller mites attached to the dorsal side of a *D. melanogaster* or *D. simulans* female dorsal abdomen. (*i–p*) Two *D. melanogaster* or *D. simulans* females with a single large mite attached to (*i–l*) the ventral or (*m–p*) dorsal side of the abdomen, grasping a cuticular fold (green) with the chelicerae (yellow) and pedipalps (blue). Scale bars indicate 500 µm if not otherwise indicated. Mite species are unidentified.

The µCT of four flies indicated that the mites are attached to flies in different ways (figure 5). The mites in figure 5*a* appear to penetrate the fly cuticle, in combination with the presence of a mite- or fly-derived substance (figure 5*d*), which is indicated by a more dense X-ray and therefore brighter contact site between mite and fly (figure 5*b*). The mites in figure 5*e* appear to attach themselves to the abdomen without cuticular penetration, visible by the bright contact site in the µCT visualization (figure 5*f*). In contrast, we hypothesize that the larger mites in figure 5*i*,*m* grasp the ventral thorax–abdomen junction and an abdominal cuticular fold with their chelicerae, respectively.

## 4. Discussion

Wounds are potential entry points for pathogens and elicit costly immune responses; therefore, they could influence host fitness. It is, therefore, important to understand the frequency of wounding in the wild so that we can understand its potential impact at the ecological scale. We find that wounding is prevalent in the wild *D. melanogaster* populations that we studied, with the abdomen being the most frequently wounded body part, particularly in females. When considering wounds in relation to interactions with other species, encapsulated parasitoid eggs were found in just under 10% of individuals. Moreover, across seven Drosophilidae species, just under 1% carried mites, and most of these mites also wounded their host. Importantly, we note that we may have under-sampled wounded or parasitized individuals because they could have lower survival, or they might be less likely to have entered our traps, compared with non-wounded and non-parasitized individuals.

Approximately 31% of *D. melanogaster* and 36% of *D. simulans* females had at least one wounded body part, which falls within estimates from studies on other insect species: for example damage or wounding was observed in less than 10% of male horned beetles (*Allomyrina dichotoma*) [49] and wounding can be inferred in up to almost 100% of two Eurasian Bluets damselflies (*Coenagrion puella* and *Coenagrion hastulatum*), as a result of ectoparasitic mite prevalence [50]. We note that non-sterile abrasion of *D. melanogaster* larval cuticle resulted in melanized marks and activated immune gene expression in the epidermis [30], so even if our study did not distinguish between penetrant and more superficial wounds, the latter may still be immunologically relevant if adults also show such a response. We found a sex-specific difference in susceptibility to cuticular injury, which might be attributed to differences in behaviour or physiology. For example, the larger body size of females might give them a higher likelihood of being wounded, and if females have a longer lifespan in the wild, they may encounter more opportunities to be wounded. However, in a study on wild *Drosophila* species, the life expectancy of adults was between 1.3 and 6.2 days, with no consistent differences between females and males [51].

The sex difference in melanized patches is driven by damage to the ventral abdomen, which often manifested in the presence of numerous small, melanized spots (figure 1*f*). We hypothesized that female abdominal melanized spots could be related to mating, through either external damage from the male or an internal physiological response to mating, although there was only a weak non-significant positive relationship between the presence of ventral abdominal wounding and copulatory wounding. Another possible explanation for the presence of melanized spots on the ventral abdomen is that this is one of the dominant mite attachment sites, a finding that aligns with other *Drosophila* studies [52,53]. We observed melanized wounds in three-quarters of the flies that had their mites removed, and wounds with a similar appearance were found in our fly wounding survey. However, the frequency with which we found mites attached to flies is considerably lower than the frequency of abdominal wounding, and given that there was no effect of sex on the presence of the mites, this cannot explain the sex difference in ventral abdominal wounds.

Given results from a laboratory study on *D. melanogaster* [45] we had hypothesized that the flies would incur wing damage, and we indeed found it in both males and females. In our study, wing damage more frequently affected an area without a vein compared with areas with veins; this could indicate that certain areas of the wing are more prone to damage than others, or it could be that damage to the veins is more likely to kill the flies or negatively affect their ability to enter the traps (however, see [54] discussed below). Collection site and season affected wing damage on veins and thorax damage, suggesting that environmental factors may play a role in determining the incidence of injuries. It has been shown that wing injuries in yellow dung flies, *Scathophaga stercoraria*, vary between seasons which may relate to an increase in male activity and/or longer female pre-reproductive periods [10]. However, we could not control for age in this study, so if older flies are more likely to be wounded than younger flies (e.g. wing damage increased with age in laboratory male *D. melanogaster* [45]), an alternative explanation is that we may have trapped flies of different ages across seasons and sites.

Because successful laboratory mating in *D. melanogaster* results in a copulatory wound [13] and we found that the prevalence of copulatory wounding in the wild varied from ~35 to ~75% across collection sites, it suggests that the numbers of virgin and mated females that we collected differed across sites. In laboratory studies, it has been shown that 80% of female *D. melanogaster* were found to reach reproductive maturity at four days post adult eclosion [55], but the first mating can also occur much earlier than this, e.g. 16.5–18 h after adult eclosion [56]. Although the time post-eclosion of the first mating may not be the same in the laboratory as in the wild owing to, for example, temperature fluctuations, the aforementioned studies suggest that we collected relatively recently emerged flies at the two sites where there was a lower frequency of copulatory wounding, or that mating opportunities were low. To give a comparative estimate from a field study carried out in southern France, 10–16% of the collected 484 female *D. melanogaster* were virgins [57] and based on the developmental speed of laboratory-reared flies, the author suggests that 22–25% of the flies that they collected were less than 24–36 h old. In the laboratory, bacteria can be transferred from male *D. melanogaster* to the female during mating, resulting in female death [58]. Therefore, if bacteria enter through genital wounds, they may negatively impact survival.

Wounds to the head, thorax and legs were less frequently observed compared with the abdomen and wings, although the higher wounding frequency to the abdomen compared with the thorax was driven by females. We only collected living flies, which must therefore have survived any injuries that they had sustained, so our study is biased towards non-fatal wounds. One possible hypothesis to explain variation in the body parts likely to be wounded, is that some wounds are more severe and result in higher mortality than others, for example the proportion of the flies that survived abdomen wounds was higher than the proportion of flies surviving thorax wounds [46]. Furthermore, in the laboratory, *D. melanogaster* can still fly stably despite receiving severe wing damage: even if up to 40% of one wing is missing, it can be compensated for by the correction of wing movements [54]. To put this value into the context of our findings, the maximum amount that we found to be missing from one wing was around 25%, with the exception of one fly, where one wing was 85–90% smaller than the other wing seemingly owing to improper post-eclosion wing expansion.

Approximately 42 hymenopteran species have been reported as endoparasitoids of *Drosophila* species [59]. In addition to wounds, we found that just under 10% of flies contained one or more melanized parasitoid eggs. Similar to estimates for the frequency of wounding, the frequency of the presence of encapsulated parasitoids in the wild is quite variable. For example, between 39 and 85% for *Leptopilina heterotoma* and *L. boulardi* in Tunisian *D. melanogaster* and *D. simulans* (Rouault 1979, referenced in [59]) and between 0 and 50% of Dutch *Drosophila* species had encapsulated parasitoids [60]. There are also numerous estimates on the proportion of encapsulating hosts in the laboratory, where unlike in the field, it is possible to know the number of cases where encapsulation was not successful, i.e. when the fly died because of the parasitoid. The proportion of encapsulating flies is also highly variable in these laboratory studies and includes a population sampled from Berlin where ~60–70% of *D. melanogaster* successfully encapsulated parasitoid eggs (e.g. [61]). We also observed variation in the prevalence of encapsulated parasitoids between seasons and locations, which aligns with previous work with wild-collected flies showing that the encapsulation rate can vary among fly populations based on geographical location or seasonal changes, influenced by factors such as host–parasitoid interactions, abiotic and biotic factors [62].

Lastly, we examined the potential for mites to cause wounds. It has been shown in *Drosophila nigrospracula* that *Macrocheles subbadius* can pierce the fly cuticle and feed on their haemolymph [63], and that mites act as parasite vectors; for instance *Varroa destructor* can transmit deformed wing virus and acute bee paralysis virus in wild honeybee colonies (*Apis mellifera*), and the endosymbiont *Spiroplasma* can be transmitted horizontally with *Macrocheles* species in *Drosophila* [28,64]. Fifteen drosophilid species including some of those that we collected, i.e. *D. melanogaster*, *D. simulans*, *D. busckii* and *D. hydei*, have been found infected with ectoparasitic mites in their natural habitats [28,53,63]. In our study, we identified three Drosophilidae species, *D. subobscura*, *D. funebris* and *S. pallida*, which to the best of our knowledge have not previously been reported to be parasitized by mites. Furthermore, two sequences had the highest similarity to two mite species, *Archidispus insolitus* and *Pergamasus* sp., which have not previously been reported on the *Drosophila* genus, in addition to the previously reported *Macrocheles* sp. [53,63]. *Archidispus* species mites have previously been reported on carabid beetles, with females exhibiting both phoretic and non-phoretic characteristics [65]. On the other hand, *Pergamasus* sp. is identified as a generalist soil predator mite, found feeding on soil-dwelling fauna, including early instar insect larvae [66]. We found mites attached to 0.7% of our collected Drosophilidae, a proportion that is remarkably consistent with the overall proportion of mites found on flies collected in Mexico and southern California using similar methods to our study [53]. It is important to note that many mite species detach themselves from their hosts once they become fully engorged such as the larvae of parasitic water mites (*Arrenurus* spp.) on zygopteran imagoes [67]; therefore we might have underestimated mite prevalence. Furthermore, if a mite load was particularly heavy, or if indeed a fly was significantly wounded, it may not have been collected by our trapping method. The µCT images suggest that at least one mite species penetrates the fly cuticle with its mouthparts, but in the other cases, there was no obvious penetration. This could be owing to there being only shallow penetration between the mite and host that cannot be captured with the methods we used, or because the species that we examined are phoretic.

Overall, the results of this study show that even in a potentially short-lived species [51] wounds are frequent in the wild. Wounding, as a common stressor, can lead to physiological changes that influence an individual’s energy allocation, reproductive success and overall survival; it not only requires a prompt and effective wound-healing process but also necessitates an efficient immune response to counter potential infections that may arise from the wounds. Additionally, the presence of ectoparasites like mites poses further challenges, as they can exacerbate the effects of wounds and contribute to disease spread. Therefore, further research is necessary to gain a comprehensive understanding of the impact of mites, other parasites, predators, competitors and conspecifics on *Drosophila* populations. Given the prevalence of wounding in our systematic study and reports from the literature on wounding in insects and other animals, it suggests that wound repair is almost certainly an important driver of the evolution of immune systems.

## Data Availability

The datasets generated in this study and the R script containing statistical analyses are available on Dryad [68]. Supplementary material is available online [69].
